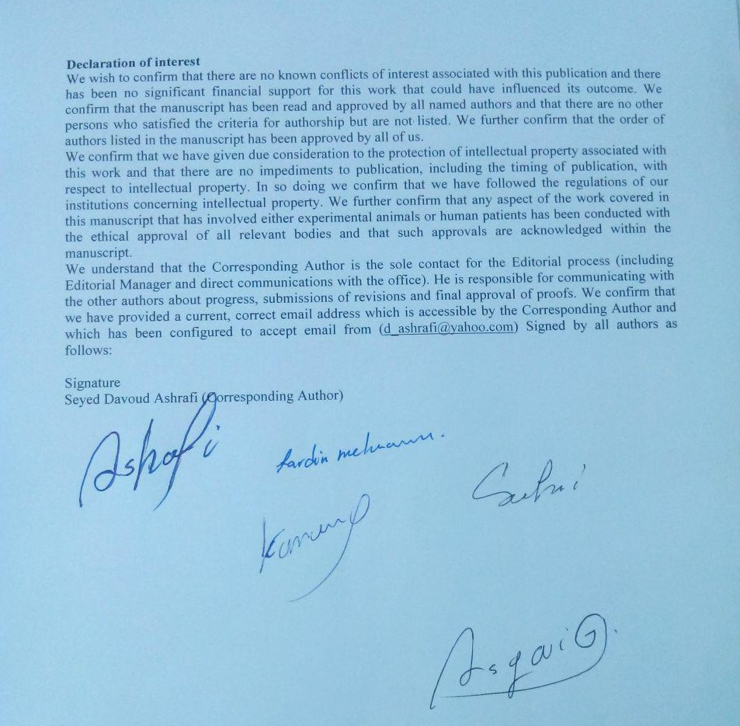# Direct Blue 71 removal from aqueous solution by laccase-mediated system; A dataset

**DOI:** 10.1016/j.dib.2018.05.056

**Published:** 2018-05-16

**Authors:** Fardin Mehrabian, Hossein Kamani, Gholam Hossein Safari, Ghorban Asgari, Seyed Davoud Ashrafi

**Affiliations:** a School of Health, Guilan University of Medical Sciences, Rasht, Iran; b Research Center of Health and Environment, Guilan University of Medical Sciences, Rasht, Iran; c Health Promotion Research Center, Zahedan University of Medical Sciences, Zahedan, Iran; d Department of Environmental Health, School of Public Health, Tabriz University of Medical Sciences, Tabriz, Iran; e Social Determinants of Health Research Center (SDHRC), Department of Environmental Health Engineering, Hamadan University of Medical Sciences, Hamadan, Iran

**Keywords:** Laccase, Dye, Direct Blue 71, Removal, Response surface method, Box–Behnken

## Abstract

The removal of Direct Blue 71 (DB71), by laccase (EC 1.10.3.2, p-bezenediol:dioxygen oxidoreductases) enzyme in presence of 2,2′-Azinobis-(3-ethylbenzothiazoline-6-sulfonate) (ABTS), in aqueous solution was investigated. Data on this article focused on the optimizing and modeling of DB71 removal by Response surface method (RSM) based on Box–Behnken design (BBD), through studying the effective variables as follows: ABTS dose (0.05–0.2 mM), Laccase dose (0.05–0.2 U mL^−1^), and pH (3–7). The results of experimental showed that laccase was able to removal DB71 with removal percentage of 83% at concentration of 50 mg L^−1^ after 30 min incubation in presence of ABTS 0.2 mM, at temperature 40 °C and pH 5. The Analysis of Variance (ANOVA) for the predicted quadratic model was done and quadratic equation showed significant *R*-Squared (0.9969), Adjusted R-Squared (0.9914) and Adequate Precision (49.7). The lack of fit is not significant due to *p*-value prob > F more than 0.05.

**Specifications Table**

**Table:** 

**Subject area**	Environmental Sciences
**More specific subject area**	Biotechnology
**Type of data**	Figure and table
**How data was acquired**	DB71 concentration; UV–vis spectrophotometer (Shimadzu UV 1700, Japan).
	pH; Digital pH meter (Metrohm).
	Temperature; Digital thermometer
	Laccase activity; Standard method by using UV–vis spectrophotometer (Shimadzu UV 1700, Japan).
**Data format**	Raw, analyzed
**Experimental factors**	Laccase activity, ABTS and pH levels were studied during the removal reaction.
**Experimental features**	The main and interaction effects of studied factors on removal efficiency were determined and the removal process was modeled.
**Data source location**	School of Health, Guilan University of Medical Sciences, Rasht, Iran.
**Data accessibility**	All data are existing.

**Value of the data**

• The data will be useful for scientific community wanting to evaluate the capability of laccase enzyme for removal of DB71 from aqueous solutions.
• The main and interaction effects of studied variables on removal efficiency of DB71 by laccase was described in this data article.
• In this data article a quadratic significant model was developed to correlate the studied variables to the DB71 removal by laccase mediated system.
• The data of successful application of laccase for DB71 removal will be useful for who wanting to design DB71 removal process from water and wastewater.

## 1 Data

**Fig. 1:**
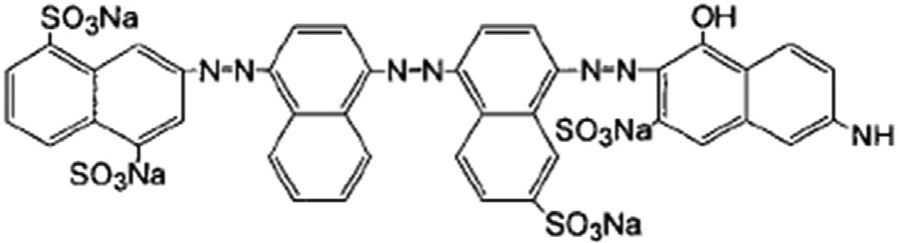
Chemical structure of DB71.

Synthetic dyes are known as an important pollutant in wastewater and environment [1], [2], [3]. The chemical structure of DB71, studied dye in this research, as a diazo synthetic dye are shown in Fig. 1. Data in Table 1 gives information about the levels of variables pH, laccase activity and ABTS dose, studied in this article. RSM based on BBD as a powerful technique [4], [5], [6], [7], was used and the experimental design matrix, along with the actual, predicted, and residual values of DB71 removal efficiency by laccase-mediated system are provided in Table 2. Table 3 gives information about the results of ANOVA, of data by using Design-expert version 7.0.0 (Stat-Ease, trial version) software and RSM based on BBD. According to the results of ANOVA main and interaction effects of all studied variables except interaction effect of BC (laccase dose to ABTS dose) are statically significant (*p*-value < 0.05). The mathematical model obtained from ANOVA was statically significant (*p*-value < 0.0001), and the quadratic equation using coded values of studied variables was as follows (Eq. (1));

(1) $$(\%)R=+63.83-6\mathrm{.5A}+5\mathrm{.37B}+10\mathrm{.38C}+1\mathrm{.75AB}-2\mathrm{.75AC}-15{\mathrm{.92A}}^{2}-1{\mathrm{.67B}}^{2}+5{\mathrm{.33C}}^{2}$$

**Table 1:** Independent factors, factors cod, actual and coded values.

**Independent factors**	**Factors cod**	**Actual and coded values**		
		**Low (− 1)**	**Medium (0)**	**High (+ 1)**
pH	A	3	5	7
Laccase dose (U mL^−1^)	B	0.05	0.125	0.2
ABTS dose (mM)	C	0.05	0.125	0.2

**Table 2:** BBD matrix of experimental run along with actual and predicted response.

**Run number**	**Levels**			**Response (%)**		
	**A**	**B**	**C**	**Actual value**	**Predicted value**	**Residuals**
1	3	0.125	0.05	47	46.625	0.375
2	7	0.125	0.05	39	39.125	− 0.125
3	5	0.2	0.05	61	62.000	− 1.000
4	5	0.05	0.05	53	52.250	0.750
5	7	0.125	0.2	54	54.375	− 0.375
6	5	0.05	0.2	73	72.000	1.000
7	3	0.125	0.2	73	72.875	0.125
8	5	0.2	0.2	83	83.750	− 0.750
9	3	0.05	0.125	48	49.125	− 1.125
10	7	0.05	0.125	32	32.625	− 0.625
11	3	0.2	0.125	57	56.375	0.625
12	7	0.2	0.125	48	46.875	1.125
13	5	0.125	0.125	63	63.833	− 0.833
14	5	0.125	0.125	64	63.833	0.166
15	5	0.125	0.125	64.5	63.833	0.666

**Table 3:** ANOVA for the obtained data and fitted quadratic model.

**Source**	**Df**	**Sum of squares**	**Mean square**	***F* value**	***p*-value Prob > *F***	**Remarks**
**A**	1	338	338	213.4	< 0.0001	Significant
**B**	1	231.1	231.1	145.9	< 0.0001	Significant
**C**	1	861.1	861.1	543.8	< 0.0001	Significant
**AB**	1	12.2	12.2	7.7	0.0388	Significant
**AC**	1	30.2	30.2	19.1	0.0072	Significant
**BC**	1	1	1	0.6	0.4628	Not significant
**A**^**2**^	1	935.4	935.4	590.7	< 0.0001	Significant
**B**^**2**^	1	10.2	10.2	6.47	0.0516	Not significant
**C**^**2**^	1	105.0	105.0	66.3	0.0005	Significant
**Model**	9	2571.9	285.7	180.4	< 0.0001	Significant
**Residual**	5	7.9	1.58			
**Lack of Fit**	3	6.7	2.2	3.8	0.2127	Not significant
**Pure Error**	2	1.1	0.5			
**Cor Total**	14	2579.9				

*R*^2^ = 0.9969 Adj *R*^2^ = 0.9914 Adeq. Precision = 49.7.

**Fig. 2:**
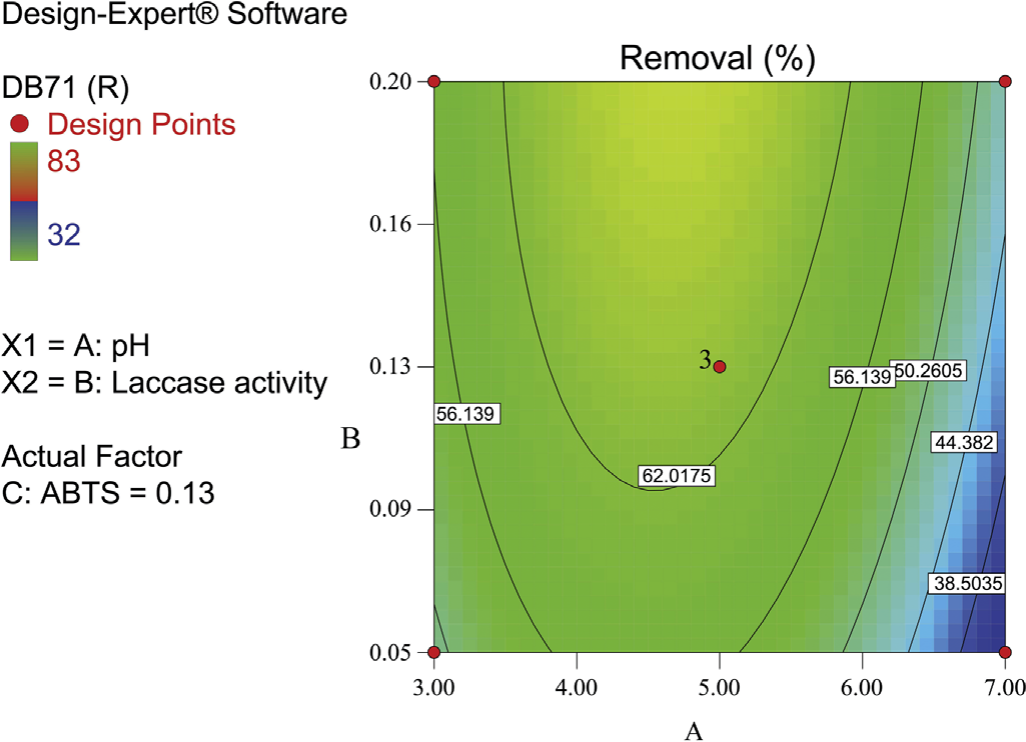
Contour plot of the interaction effects of pH (A) and laccase dose (B) on DB71 removal.

**Fig. 3:**
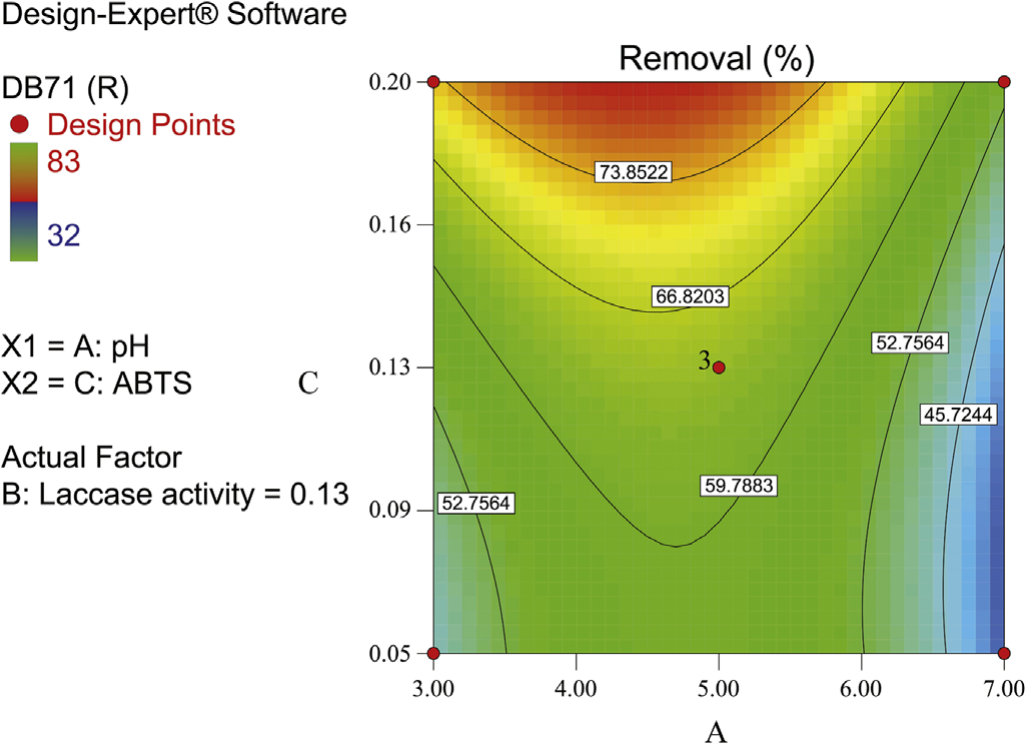
Contour plot of the interaction effects of pH (A) and ABTS dose (C) on DB71 removal.

**Fig. 4:**
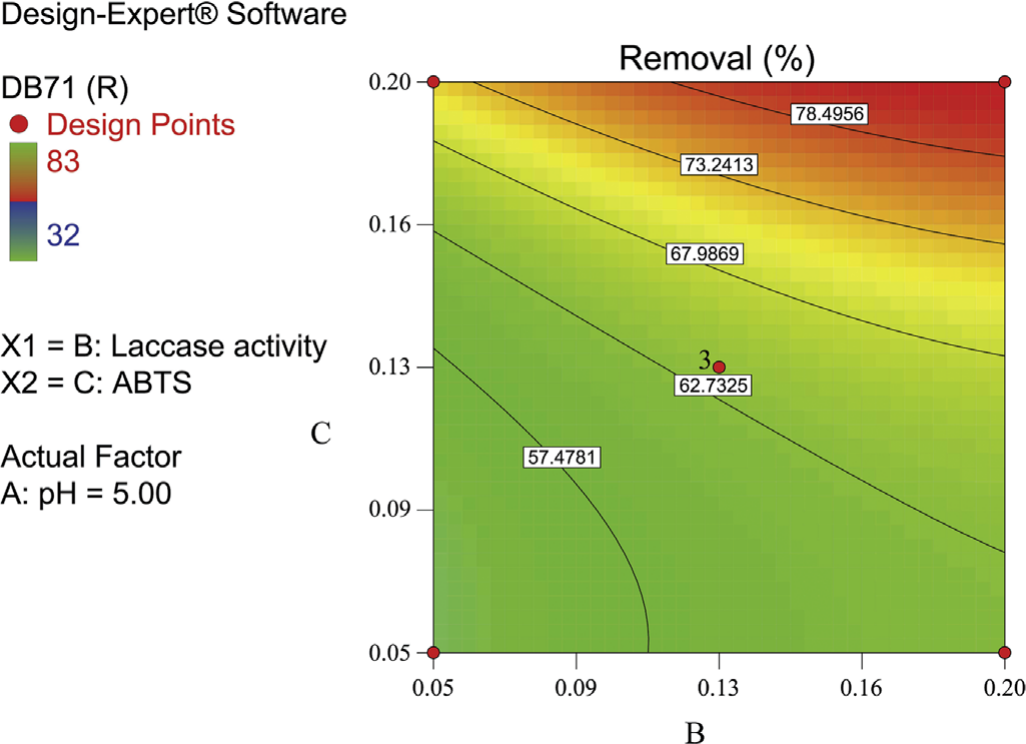
Contour plot of the interaction effects of laccase dose (B) and ABTS dose (C) on DB71 removal.

**Fig. 5:**
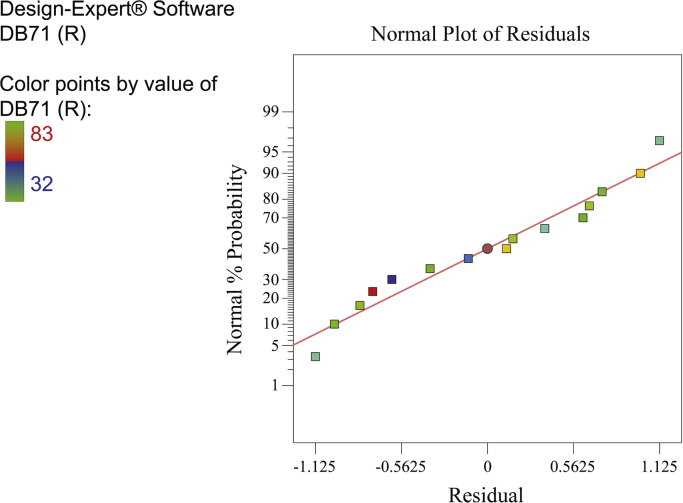
Normal probability plot of residual values.

**Fig. 6:**
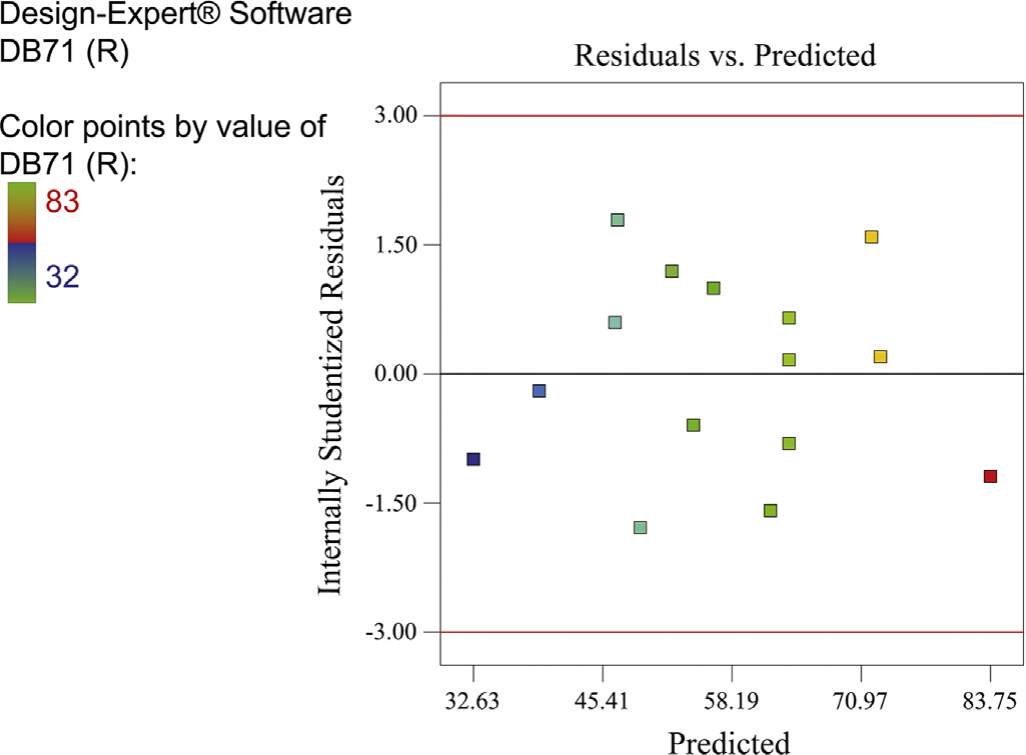
Plot of residual values vs. predicted response values.

**Fig. 7:**
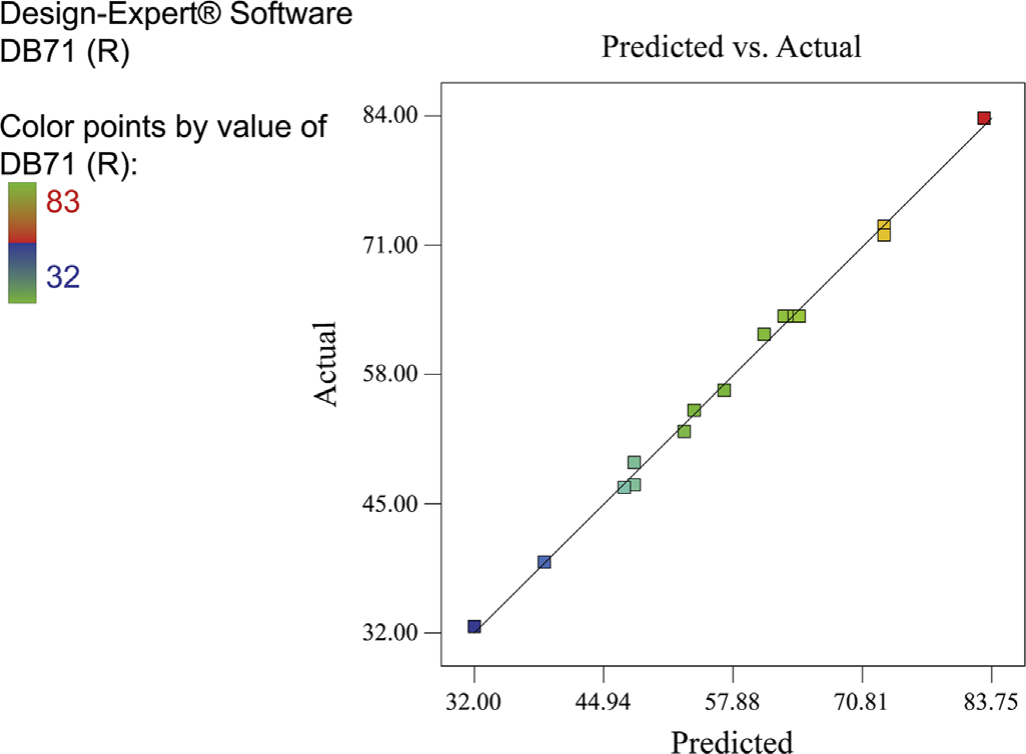
Actual values of removal efficiency and the predicted values from the mathematical model.

Fig. 2, Fig. 3, Fig. 4 show the contour plot of the interaction effects of pH, laccase dose and ABTS dose on DB71 removal by laccase enzyme. Fig. 5 shows the normal probability plot of the residual values. Plot of the residual values vs. predicted response values are shown in Fig. 6. Fig. 7 shows the actual values of removal efficiency and the predicted values from the mathematical model.

## 2 Experimental design, materials and methods

### 2.1 Materials and analytical measurements

All of the chemicals were of the highest purity available. Laccase from *Trametes versicolor* (activity > 10 U mg^−1^) and ABTS were purchased from Sigma Aldrich (St. Louis, MO, USA). DB71 was purchased from Alvan Sabet Co. (Tehran, Iran). For assaying the laccase activity, oxidation of ABTS was monitored according to standard method which described in previous works [5], [8], [9]. Briefly, one activity unit of laccase was defined as the amount of laccase that oxidize 1μmol of substrate like ABTS per min. Dye concentration was measured by using UV–vis spectrophotometer (Shimadzu UV 1700, Japan) through a calibration curve by analysis the maximum absorbance wavelength 584 nm and the removal percentage was then calculated [8], [10].

### 2.2 Removal experiments and ANOVA analyzing

Experimental design was developed using RSM based on Box-Behnken design by Design-expert software to determine the runs levels of the studied variables in this research. In order to evaluate the main and interaction effects of variables on DB71 removal and to develop a mathematical model for removal process the ANOVA analyzing was used. The DB71 removal study was done according to the method previously described [8]. Briefly, the final volume of reaction solutions were 5 mL which prepared in 0.1 M citrate sodium buffer. According to the variable matrix (Table 2), the removal reaction was started by adding laccase and incubated at 40 °C and 150 rpm for 30 min.

## Figures and Tables

**Supplementary Fig. S1:**